# Evolution of Multicopper Oxidase Genes in Coprophilous and Non-Coprophilous Members of the Order Sordariales

**DOI:** 10.2174/138920211795564368

**Published:** 2011-04

**Authors:** Stefanie Pöggeler

**Affiliations:** Department of Genetics of Eukaryotic Microorganisms, Institute of Microbiology and Genetics, Georg-August University Göttingen, Grisebachstr. 8, 37077 Göttingen, Germany

**Keywords:** Laccase, coprophilous fungi, Sordariales, *Sordaria macrospora*, *Podospora anserina*, *Neurospora crassa*, *Chaetomium globosum*, gene duplication.

## Abstract

Multicopper oxidases (MCO) catalyze the biological oxidation of various aromatic substrates and have been identified in plants, insects, bacteria, and wood rotting fungi. In nature, they are involved in biodegradation of biopolymers such as lignin and humic compounds, but have also been tested for various industrial applications. In fungi, MCOs have been shown to play important roles during their life cycles, such as in fruiting body formation, pigment formation and pathogenicity. Coprophilous fungi, which grow on the dung of herbivores, appear to encode an unexpectedly high number of enzymes capable of at least partly degrading lignin. This study compared the MCO-coding capacity of the coprophilous filamentous ascomycetes *Podospora anserina* and *Sordaria macrospora* with closely related non-coprophilous members of the order Sordariales. An increase of MCO genes in coprophilic members of the Sordariales most probably occurred by gene duplication and horizontal gene transfer events.

## 1. INTRODUCTION

Multicopper oxidases (MCOs) are a family of enzymes that contain copper atoms in their catalytic center, and are capable of coupling the oxidation of a substrate, e.g. polyphenols, aromatic amines and a variety of other components, with a four-electron reduction of molecular oxygen to water [1]. A large group within the MCO family are laccases typically found in plants and fungi (benzendiol:oxygen oxidoreductase EC 1.10.32) [2]. Other members of the MCO family are ferroxidases (EC1.163.1) ascorbate oxidases (EC1.103.3), bilirubin oxidases, and cerloplasmin, found in vertebrate [3].

While plant laccases mainly participate in lignin polymer formation, fungal laccases are involved in the degradation of lignin and humic acids, but also have important roles in developmental processes such as fruiting body development and pigmentation. Because they oxidize, polymerize, or transform phenolic or anthropogenic compounds into less toxic derivatives, fungal laccases have been used for various biotechnological applications such as food processing, bioremediation of waste water, and removal of lignin from wood fibers [4-7]. To date, predominately the laccases of wood-rotting basidiomycetes have been described and used for biotechnological applications. Although much better tractable genetically than the basidiomycetes, the laccases of ascomycetes have been used to a much lesser extent in biotechnology [2, 8]. Nevertheless, several laccases of filamentous ascomycetes have been purified and characterized, for example, from the plant pathogens *Magnaporthe grisea* [9], *Ophiostoma novo-ulmi* [10] and *Gaeumannomyces graminis* [11, 12] as well as from soil ascomycete species such as *Aspergillus nidulans* [13] and *Penicillium chrysogenum* [14] and fresh water ascomycetes [15]. In addition, laccases have been analyzed from lignocellulolytic ascomycetes such as *Stachybotrys chartarum* [16] and *Trichoderma reesei* [17] and in wood-colonizing *Xylaria* species [18]. The three-dimensional structure of the laccases from the thermophilic ascomycete *Melanocarpus albomyces* has been solved as one of the first complete laccase structures [19, 20].

Analysis of the genome of the coprophilous fungus *Podospora anserina* (order Sordariales) revealed an unexpectedly large number of putative ligin-degradating enzymes, among them several laccases [21, 22]. In nature *P. anserina* lives exclusively on the dung of herbivores and usually fructifies at the late stage of dung decomposition when simple carbohydrates are depleted. The genome of *P. anserina* evolved a more comprehensive coding capacity for enzymes that degrade complex biopolymers than the close relative *Neurospora crassa*, which in nature is often found on scorched vegetation after wildfires or agricultural burns [22-24]. The genome of an even closer relative of *P. anserina*, *Chaetomium globosum* is also publicly available (http://www.broadinstitute.org/annotation/genome/chaetomium_globosum). Recently, the genome sequence of another coprophilous member of the Sordariales, *Sordaria macrospora*, was published [25]. Similar to *P. anserina*, *S. macrospora* in nature lives exclusively on the dung of herbivores and also fructifies at the late stages of dung decomposition [26]. *C.  globosum* is a wood-destroying fungus causing the so-called mildew-rot [26]. It is frequently isolated from water-damaged buildings and is associated with sick building syndrome, a set of nonspecific symptoms resulting from poor indoor air quality [27]. In the 1970s, extracellular laccases of *N. crassa* and *P. anserina* were purified to apparent homogeneity by classical purification techniques [28, 29] Furthermore, it has been demonstrated that *P. anserina* produce multiple laccases isoenzymes [29]. Using molecular biology techniques, later some of the respective laccase genes of *N. crassa* and *P. anserina* were cloned [30, 31]. The availability of the genome sequences of coprophilous and non-coprophilous members of the genus Sordariales makes it now possible to compare their MCO coding capacity and determine if the increased variety of this class of enzymes is linked to a coprophilous lifestyle.

The data presented here revealed gene duplication, acquisition and gene loss events in the evolutionary history of MCO genes in the genomes of Sordariales members. An increase in MCO genes in coprophilic members most probably occurred by gene duplication and horizontal gene transfer events and might contribute to their ability to grow in an extremely competitive habitat.

## 2. MATERIALS AND METHODS

### 2.1. Sequence Analysis

Fungal genomic sequences used for this study are available at: Fungal Genome Initiative (Broad Institute: http://www.broad.mit.edu/annotation/fgi/), (http://podospora. igmors.u-psud.fr) and the Institut für Allgemeine und Molekulare Botanik Ruhr-Universität Bochum, 44780 Bochum, Germany [25] All downloads were performed before 1 December 2010.

To identify laccases and other multi-copper oxidases of *C. globosum*, *N. crassa*, *P. anserina* and *S. macrospora* blastp and tblastn [32] searches with the protein sequences of the laccases MaL from *Melanocarpus albomyces* (Q70KY3) as query, and key word searches were performed. Annotations of several laccases were found to be incorrect, because conserved domains were not detectable under the given annotated genes or accession numbers. Because often domains could be found within the accordant open reading frames or in the sequence of the annotated gene, the gene locus is given in Results and Discussion instead of the accession numbers.

### 2.2. Phylogenetic Analysis

Multiple protein sequence alignments were performed using the clustalX program [33]. Phylogenetic analysis was made with programs from package PHYLIP version 3.6 (http://evolution.genetics.washington.edu/phylip.html). Distance matrices were calculated using program PROTDIST and were then used for constructing trees with the neighbor-joining program NEIGHBOR. Statistical significance was evaluated by bootstrap analysis with 1000 iterations of bootstrap samplings generated with SEQBOOT. A majority rule consensus tree was subsequently generated using the program CONSENSE. The consensus trees were graphically displayed using the program TreeView (Win 32) 1.6.6 [34] and saved for graphical representation using Adobe Illustrator. Phylogenetic trees were generated based on an alignment that starts with Gly^125^ and ends with Pro^209^ of the Laccase precursor NCU04528.4 from *N. crassa* (XP_956939.1) [35]. This region includes laccase L1 and L2 signature sequences [36]. For the phylogenetic analysis of the L1-L2 region, modifications concerning intron splicing of an annotated laccase genes was made to increase the sequence identity to related laccases from other members of the Sordariales (Fig. **S1**).

### 2.3. Prediction of Secretion Signals and Transmembrane Domains

The online programs SignalP was used to determine cleavage sites of putative signal peptides [37]. Programs and TMHMM (http://www.cbs.dtu.dk/services/TMHMM-2.0/) and HMMTOP [38, 39] were used to predict transmembrane domains.

## 3. RESULTS AND DISCUSSION

### 3.1. Multiple MCOs are Encoded in Coprophilous and Non-Coprophilous Members of the Sordariales

To compare the MCO coding capacity of coprophilous and non-coprophilous members of the fungal order Sordariales, the genomes of *N. crassa*, *S. macrospora*, *P. anserina* and *C. globosum* were mined using blastp and tblastn searches with the protein sequence of the MaL laccase of the thermophilic ascomycete *M. albomyces*, and with keyword searches. A total of 49 MCO amino acid sequences were obtained from the genomic databases of *N. crassa*, *S. macrospora*, *P. anserina* and *C. globosum*, including putative laccases, ascorbate oxidases, bilirubin oxidases and ferroxidases (Table **1**). The highest number of MCOs was identified in the coprophilous fungi *S. macrospora* and *P. anserina* (15 MCO genes), whereas *N. crassa* and *C. globosum* encoded only 11 and 8 MCOs, respectively. Based on the analyses of more than 100 laccases, four ungapped sequence regions, L1-L4, have been identified as the overall signature sequences for distinguishing laccases within the broader class of MCOs [40]. In the four fungi analyzed, most of the identified MCOs belong to the laccase group. Two putative laccases of *S. macrospora* (SMAC09721 and SMAC03318) and one of *C. globosum* (CHGG_08215) lack the L3 and L4 signature in the predicted sequence. This could be due to annotation and sequence errors or the identified genes could encode non-functional pseudogenes. Consistent with an extracellular function established for other well-characterized fungal laccases, including laccases of the basidiomycete *Coprinopsis cinerea* [41] and *N. crassa* [31] and *M. albomyces* [19], signal peptides were predicted for most analyzed laccases (Table **1**) Signal peptides length were 17- 32 amino acids (Table **1**). However, at least one isoform was present in each fungus that was predicted to contain no signal peptide. This again may be due to annotation errors or members of the order Sordariales may contain intracellular laccase isoforms. Intercellular laccase activity has been identified in basidiomycetes and ascomycetes, and is proposed to be involved in the transformation of low molecular weight phenolic compounds [1]. Five intracellular laccase isoforms were previously shown to be produced by the basidiomycete *Pleurotus ostreatus* when co-cultivated with the ascomycete *Trichoderma longibrachiatum,* suggesting expression of these enzymes can be induced by interspecies interactions [42]. Thus, the fungi analyzed here may possess intracellular laccases induced under specific environmental conditions.

Consistent with previous studies by Espagne *et al.* [22] and Hoegger *et al.* [2], eight putative laccases were identified in *N. crassa*. In contrast to Espagne *et al*. [22], only nine putative laccases were found in *P. anserina*. The putative laccase Pa_1_16470 was not identified in this analysis. However, Pa_6_2550 was predicted as an MCO since it contains all of the conserved laccase signature sequences. As in Hoegger *et al.* [2], four laccases were found in *C. globosum* and one C-terminally truncated (CHGG11082.1) laccase lacking L3 and L4. *S. macrospora* encodes at least nine putative laccases and two C-terminally truncated laccase-like proteins without the L3 and L4 domains.

Each of the four fungi analyzed here encodes two homologs of the *Saccharomyces cerevisiae* plasma membrane ferroxidase Fet3p. In *S. cerevisiae*, Fet3p receives iron(II) ions from cell-surface iron reductases and passes iron(III) ions to the iron permease Ftr1p [43, 44]. Similar to other filamentous ascomycetes [2], one of the *fet3* homologs of the Sordariales is located directly downstream of a *ftr1* homolog (Table **1**). With the exception of *C. globosum* (CHGG08215.1), only Fet3p homologs clustered with *ftr1* (NCU03498.4, SMAC_07233, Pa_6_4220) are predicted to have one transmembrane domain (Table **1**). Thus, these homologs may have a similar function as in *S. cerevisiae*, while the other seems to be an extracellular enzyme and may fulfill another role.

In addition to laccases and ferroxidases, *S. macrospora*, *P. anserina* and *C. globosum* encode a putative ascorbate oxidase. This enzyme is highly specific for the reducing substrate, ascorbate, and other compounds with a lactone ring with an enediol group adjacent to a carbonyl group [45]. In plants, ascorbate oxidases modulate the redox state of the apoplastic ascorbate pool and thereby regulate defense and growth [46]. The function of fungal ascorbate oxidase is so far unknown.

Except for *C. globosum,* the fungi analyzed here encode at least one bilirubin oxidase. This class of MCO oxidizes bilirubin to biliverdin. Putative bilirubin oxidases from *N. crassa* (NCU05042.4), *S. macrospora* (SMAC_07604) and *P. anserina* (Pa_6_11170 and Pa_5_1710) are highly similar to the bilirubin oxidase from the ascomycete *Myrothecium verrucaria* (BAA03166) [47], which is the best characterized fungal bilirubin oxidase [48]. Interestingly, the *M. verrucaria* bilirubin oxidase was identified in a screen for microorganisms decolorizing urine and feces in raw sewage [49]. *M. verrucaria* was also identified on the dung of horses [50]. Thus the coprophilous fungi, *S. macrospora* and *P. anserina* seem to have the ability to oxidize bilirubin, the degradation product of hemoglobin, which is mainly excreted *via* feces.

### 3.2. Phylogenetic Analysis of MCOs from Coprophilous and Non-Coprophilous Members of the Sordariales

**Fig. (1).**
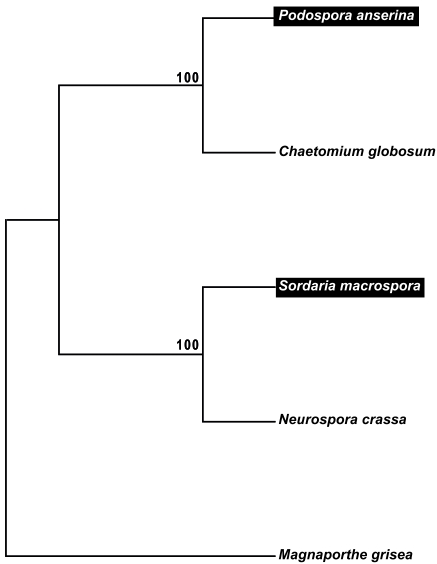
Bootstrap consensus tree from a neighbor joining analysis of coprophilous and non-coprophilous members of the Sordariales and *Magnaporthe grisea* (Magnaporthales) based on three conserved genes. The bootstrap values from 1000 replicates are shown if they exceed 50%. Accession numbers: *Neurospora crassa* (XP_956977.1, glyceraldehyde-3-phosphate dehydrogenase; AAA33617.1, tubulin beta chain; CAE76188.1, translation elongation factor 1-alpha); *Sordaria macrospora* (CAC86412.2, glyceraldehyde-3-phosphate dehydrogenase; CBI53599.1, tubulin beta chain; CAA65435.1, translation elongation factor 1-alpha); *Podospora anserina* (XP_001909301.1, glyceraldehyde-3- phosphate dehydrogenase; XP_001906071.1, tubulin beta chain; CAA52806.1, elongation factor 1-alpha); *Chaetomium globosum* (XP_001225636.1, glyceraldehyde-3-phosphate dehydrogenase; XP_001226966.1, tubulin beta chain; EAQ89925.1 elongation factor 1-alpha); *Magnaporthe grisea* (XP_368160.1, glyceraldehyde-3-phosphate dehydrogenase; XP_368640.1, tubulin beta chain; EDJ94428.1, elongation factor 1-alpha). The tree was rooted with *M. grisea.* Coprophilous  *S. macrospora* and *P. anserina* are indicated in white and boxed in black.

Previous studies identified that *S. macrospora* is a close relative of *N. crassa*. Both fungi exhibit a 90% nucleic acid identity within coding regions of orthologous genes, as well as a high degree of synteny over large genomic regions [25, 51]. Within protein-coding regions, *S. macrospora* and *N. crassa* share ∼95% amino acid identity (similar to mice and humans) [52]. *P. anserina* was shown to be more distantly related to *N. crassa* and *S. macrospora*, but more closely related to *C. globosum* [22, 53]. To verify this proposed relationship among the four members of the order Sordariales, a ClustalX amino acid alignment of the conserved proteins elongation factor 1-alpha, glyceraldehyde-3-phosphate dehydrogenase and β-tubulin was used for a neighbor-joining analysis of 1000 bootstrapped datasets. The consensus tree verified the close relationship of *S. macrospora* and *N. crassa* as well as of *P. anserina* and *C. globosum* (Fig. **1**).

**Fig. (2).**
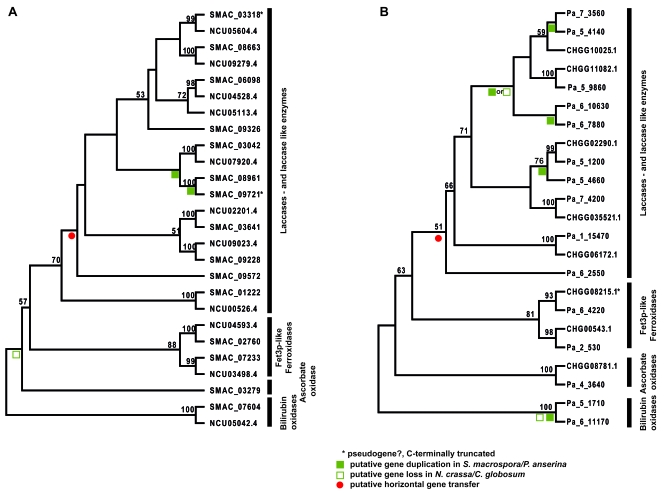
Bootstrap consensus tree from a neighbor joining analysis of partial MCO amino acid sequences corresponding to L1-L2 region. The bootstrap values from 1000 replicates are shown at the nodes if they exceed 50%. (**A**) Phylogenetic analysis of *N. crassa* and *S. macrospora* MCOs. (**B**) Phylogenetic analysis of *C. globosum* and *P .anserina* MCOs. Abbreviations and description of proteins see Table **1**.

**Fig. (3).**
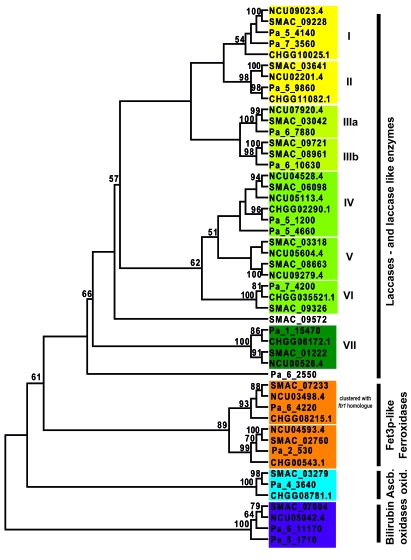
Combined bootstrap consensus tree from a neighbor joining analysis of partial MCO amino acid sequences corresponding to L1-L2 region. The bootstrap values from 1000 replicates are shown at the nodes if they exceed 50%. Abbreviations and description of proteins see Table **1**.

To analyze whether gains, duplications and losses of MCO genes occurred when two closely related fungi inhabited different ecological niches, a phylogenetic analysis was performed using the identified MCO proteins of *N. crassa* and *S. macrospora* and of *P. anserina* and *C. globosum*. The neighbor-joining trees shown in Fig. (**2**) are based on a ClustalX alignment of a protein region spanning the laccase signature sequences L1-L2 [36]. Fig. (**2a**) shows that two putative gene duplication events and the gain of one MCO gene by a putative horizontal gene transfer event may have occurred in *S. macrospora*. These events may have led to the large laccase gene family in *S. macrospora*. However, note that the annotated ORFs SMAC_03318 and SMAC09721 encode C-terminally truncated proteins without laccase domains L3 and L4. Thus, these paralogs may be pseudogenes that resulted from a gene duplication event and subsequent loss of function or these ORFs may be wrongly annotated. No *N. crassa* ortholog could be identified for SMAC_09572. A BLAST analysis of SMAC_09572 revealed a putative MCO of the necrotrophic fungal phytopathogen *Sclerotinia sclerotiorum* order Helotiales (XP_001594389, 1e^-137^) as the closest ortholog. Thus, *SMAC_09572* may have been acquired by horizontal gene transfer. Also previous studies revealed that *S. macrospora* contains more polyketide biosynthesis genes than *N. crassa* and phylogenetic analyses suggest that some of these genes may have also been acquired by horizontal gene transfer from a distantly related ascomycete group [25]. Furthermore, phylogenetic analysis of *N. crassa* and *S. macrospora* MCOs revealed that *N. crassa* may have lost a gene coding for an ascorbate oxidase, which is not only present in *S. macrospora* but also in *C. globosum* and *P. anserina* (Figs. **2** and **3**).

Similar to *S. macrospora*, several laccase gene duplication events and duplication of the bilirubin oxidase gene may have occurred in the coprophilous fungus *P. anserina* (Fig. **2b**). *C. globosum* may have lost the bilirubin oxidase gene and perhaps one laccase gene. No *C. globosum *ortholog could be identified for *Pa_6_2550*. A BLAST analysis of the encoded protein identified a putative MCO of the plant pathogen *Glomerella graminicola *order Hypocreales (EFQ26167, 3e^-132^) as the closest ortholog. Thus, similar *S. macrospora* *P. anserina* seems to have acquired one additional laccase gene by horizontal gene transfer. Previously horizontal transfer of a mitochondrial plasmid from the discomycete *Ascobolus immersus* to *P. anserina* was reported [54]. The combined phylogenetic analysis of all MCOs (Fig. **3**) revealed a strict separation of bilirubin oxidases, ascorbate oxidases, ferroxidases and laccases. According to the phylogenetic tree seven different groups of laccases may be distinguished. Group I, II, IV and VII comprises laccases orthologs of all four species. Not surprisingly, laccases SMAC0572 of *S. macrospora* and Pa_6_2550 of *P. anserina*, presumed to have been acquired by horizontal gene transfer, do not cluster within these groups. Laccase group IV contains Pa_5_1200 and NCU4529.4, which have been demonstrated to be active laccase enzymes [29-31]. Laccases from this group (NCU04528.4; SMAC_06098, CHGG02290.1, Pa_5_1200) are demonstrated or predicted to be C-terminally processed. C-terminal processing is also predicted for laccases of group VI (Fig. **3** and Table **1**). Laccase group III contains mainly laccases from the coprophilous fungi *S. macrospora* and *P. anserina* and may be subdivided into two subgroups. Group VII consist of putative intracellular isoforms of laccases enzymes laccases without N-terminal signal sequence (Table **1**). Fet3-like ferroxidases comprise two subgroups, with each containing homologs of all four species. One subgroup consists of *fet3* homologs located adjacent to a putative iron permease *ftr1* homolog, while the other subgroup is not clustered with a permease gene (Fig. **3** and Table **1**). As already seen in Fig. (**2**), *N. crassa* lacks an ascorbate oxidase and *C. globosum* does not encode a bilirubin oxidase.

## 4. CONCLUSION

Closely related members of the order Sordariales which inhabit different ecological niches exhibit different coding capacities for MCOs. The analysis of the phylogeny of MCO gene families in coprophilous and non-coprophilous members of the order Sordariales revealed that the laccase gene family is particularly large in coprophilous fungi. The increase of laccase genes in the coprophilous fungi *S. macrospora* and *P. anserina* can be attributed to horizontal gene transfer and gene duplication events. While the complete set of MCO genes was maintained in coprophilous fungi some MCOs disappeared in their closely related non-coprophilous relatives, *N. crassa* and *C. globosum*.

## SUPPLEMENTARY MATERIAL

Supplementary material is available on the publishers Web site along with the published article.

## Figures and Tables

**Table 1.** Characteristics of MCOs Identified in Members of the Order Sordariales

Species	Multicopper oxidase gene ID	Length of the precursor aa	Secretion (size in aa of the signal peptide predicted by Signal P/Signal P probability)	MCO classification
*Neurospora crassa*				
	NCU04528.4^1^,^3^	619	+, (21/1.000)	laccase
	NCU09279.4	601	+ (20/1.000)	laccase
	NCU05604.4	607	+ (22/1.000)	laccase
	NCU05113.4	595	+, (19/1.000)	laccase
	NCU02201.4	588	+ (18/0.999)	laccase
	NCU00526.4	604	-	laccase
	NCU09023.4	700	+, (23/0.982)	laccase
	NCU07920.4	739	+ (22/0.897)	laccase
	NCU03498.4	693	+, (19/0.999)	Fet3 ferroxidase^4^,^6^
	NCU04593.4	544	+, (19/1.000)	Fet3 ferroxidase
	NCU05042.4	620	+, (27/0.998)	bilirubin oxidase
*Sordaria macrospora*				
	SMAC_06098^3^	598	+, (21/1.000)	laccase
	SMAC_08663	597	+, (20/1.000	laccase
	SMAC_03641	597	+, (18/1.000	laccase
	SMAC_08961	577	+, (20/0.997)	laccase
	SMAC_03318^5^	507	+, (22/1.000)	laccase
	SMAC_01222	602	-	laccase
	SMAC_09228	593	+, (23/0.990)	laccase
	SMAC_09572	559	+, (24/0.987)	laccase
	SMAC_03042	751	+, (22/0.907)	laccase
	SMAC_09326^3^	614	+, (25/0.912)	laccase
	SMAC_09721^5^	241? C-terminally truncated	+, (20/0.998)	laccase
	SMAC_07233	704	+, (18/0.976)	Fet3 ferroxidase^4^,^6^
	SMAC_02760	564	+, (20/1.000)	Fet3 ferroxidase
	SMAC_03279	680	+, (22/0.996)	ascorbate oxidase
	SMAC_07604	643	+, (22/0.999)	bilirubin oxidase
*Podospora anserina*				
	Pa_7_4200^3^	610	+, (22/0.998)	laccase
	Pa_5_1200^2^,^3^	621	+, (23/1.000)	laccase
	Pa_5_4660r	621	+, (32/0.987)	laccase
	Pa_5_9860	597	+, (19/0.999)	laccase
	Pa_6_10630	568	+, (19/0.995)	laccase
	Pa_5_4140	675	+, (17/0.943)	laccase
	Pa_7_3560	641	-	laccase
	Pa_1_15470	594	-	laccase
	Pa_6_7880	758	+?, (26/0.298)	laccase
	Pa_6_2550	695	-	multicopper oxidase
	Pa_2_530	575	+, (20/1.000)	Fet3 ferroxidase
	Pa_6_4220	674	+, (28/0.994)	Fet3 ferroxidase^4^,^6^
	Pa_4_3640	666	+, (20/0.999)	ascorbate oxidase
	Pa_6_11170	595	+, (20/0.999	bilirubin oxidase
	Pa_5_1710	625	-	bilirubin oxidase
*Chaetomium globosum*				
	CHGG035521.1^3^	612	+, (24/0.992)	laccase
	CHGG02290.1^3^	619	+, (21/1.000)	laccase
	CHGG11082.1^5^	539	+, (17/0.995)	laccase
	CHGG10025.1	618	+, (23/0.979)	laccase
	CHGG06172.1	595	-	laccase
	CHG00543.1	602	+, (21/0.971)	Fet3 ferroxidase
	CHGG08215.1	450	+, (21/0.873)	Fet3 ferroxidase^4^
	CHGG08781.1	645	+, (20/0.996)	ascorbate oxidase

1 laccase activity has been demonstrated [31].

2 laccase activity has been demonstrated [29, 30].

3 denotes putative or experimentally verified additional C-terminal processing.

4 clustered with *S. cerevisiae* ftr1 homologue.

5 lacking L3 and L4 signature sequence in the predicted sequence.

6 one TM domain predicted by the TMHMM Server v. 2.0 and HMMTOP.

## References

[1] Baldrian P (2006). Fungal laccases – occurrence and properties. FEMS Microbiol. Rev.

[2] Hoegger PJ, Kilaru S, James TY, Thacker JR, Kües U (2006). Phylogenetic comparison and classification of laccase and related multicopper oxidase protein sequences. FEBS J.

[3] Sakurai T, Kataoka K (2007). Basic and applied features of multicopper oxidases, CueO, bilirubin oxidase, and laccase. Chem. Rec.

[4] Brijwani K, Rigdon A, Vadlani PV (2010). Fungal laccases: production, function, and applications in food processing. Enzyme Res.

[5] Riva S (2006). Laccases: blue enzymes for green chemistry. Trends Biotechnol.

[6] Majeau J-A, Brar SK, Tyagi RD (2010). Laccases for removal of recalcitrant and emerging pollutants. Bioresource Technol.

[7] Rodríguez Couto S, Toca Herrera JL (2006). Industrial and biotechnological applications of laccases: A review. Biotechnol. Adv.

[8] Rodgers CJ, Blanford CF, Giddens SR, Skamnioti P, Armstrong FA, Gurr SJ (2010). Designer laccases: a vogue for high-potential fungal enzymes?. Trends Biotechnol.

[9] Iyer G, Chattoo BB (2003). Purification and characterization of laccase from the rice blast fungus, *Magnaporthe grisea*. FEMS Microbiol. Lett.

[10] Binz T, Canevascini G (1997). Purification and partial characterization of the extracellular laccases from *Ophiostoma novo-ulmi*. Curr. Microbiol.

[11] Edens WA, Goins TQ, Dooley D, Henson JM (1999). Purification and characterization of a secreted laccase of *Gaeumannomyces graminis var. tritici*. Appl. Environ. Microbiol.

[12] Litvintseva AP, Henson JM (2002). Cloning, characterization, and transcription of three laccase genes from *Gaeumannomyces graminis var. tritici*, the take-all fungus. Appl. Environ. Microbiol.

[13] Scherer M, Fischer R (1998). Purification and characterization of laccase II of *Aspergillus nidulans*. Arch Microbiol.

[14] Rodríguez A, Falcón MA, Carnicero A, Perestelo F, la Fuente GD, Trojanowski J (1996). Laccase activities of *Penicillium chrysogenum* in relation to lignin degradation. Appl. Microbiol. Biotechnol.

[15] Martin C, Corvini PFX, Vinken R, Junghanns C, Krauss G, Schlosser D (2009). Quantification of the influence of extracellular laccase and intracellular reactions on the isomer-specific biotransformation of the Xenoestrogen technical Nonylphenol by the aquatic hyphomycete *Clavariopsis aquatica*. Appl. Environ. Microbiol.

[16] Mander GJ, Wang H, Bodie E, Wagner J, Vienken K, Vinuesa C, Foster C, Leeder AC, Allen G, Hamill V, Janssen GG, Dunn-Coleman N, Karos M, Lemaire HG, Subkowski T, Bollschweiler C, Turner G, Nusslein B, Fischer R (2006). Use of laccase as a novel, versatile reporter system in filamentous fungi. Appl. Environ. Microbiol.

[17] Levasseur A, Saloheimo M, Navarro D, Andberg M, Pontarotti P, Kruus K, Record E (2010). Exploring laccase-like multicopper oxidase genes from the ascomycete *Trichoderma reesei*: a functional, phylogenetic and evolutionary study. BMC Biochem.

[18] Liers C, Ullrich R, Steffen K, Hatakka A, Hofrichter M (2006). Mineralization of ^14^C-labelled synthetic lignin and extracellular enzyme activities of the wood-colonizing ascomycetes *Xylaria hypoxylont;* and *Xylaria polymorpha*. Appl. Microbiol. Biotechnol.

[19] Hakulinen N, Kiiskinen L-L, Kruus K, Saloheimo M, Paananen A, Koivula A, Rouvinen J (2002). Crystal structure of a laccase from *Melanocarpus albomyces* with an intact trinuclear copper site. Nat. Struct. Mol. Biol.

[20] Hakulinen N, Andberg M, Kallio J, Koivula A, Kruus K, Rouvinen J (2008). A near atomic resolution structure of a *Melanocarpus albomyces* laccase. J. Struct. Biol.

[21] Paoletti M, Saupe S (2008). The genome sequence of *Podospora anserina*, a classic model fungus. Genome Biol.

[22] Espagne E, Lespinet O, Malagnac F, Da Silva C, Jaillon O, Porcel B, Couloux A, Aury J-M, Segurens B, Poulain J, Anthouard V, Grossetete S, Khalili H, Coppin E, Dequard-Chablat M, Picard M, Contamine V, Arnaise S, Bourdais A, Berteaux-Lecellier V, Gautheret D, de Vries R, Battaglia E, Coutinho P, Danchin E, Henrissat B, Khoury R, Sainsard-Chanet A, Boivin A, Pinan-Lucarre B, Sellem C, Debuchy R, Wincker P, Weissenbach J, Silar P (2008). The genome sequence of the model ascomycete fungus *Podospora anserina*. Genome Biol.

[23] Raju NB (2009). Neurospora as a model fungus for studies in cytogenetics and sexual biology at Stanford. J. Biosci.

[24] Galagan JE, Calvo SE, Borkovich KA, Selker EU, Read ND, Jaffe D, FitzHugh W, Ma L-J, Smirnov S, Purcell S, Rehman B, Elkins T, Engels R, Wang S, Nielsen CB, Butler J, Endrizzi M, Qui D, Ianakiev P, Bell-Pedersen D, Nelson MA, Werner-Washburne M, Selitrennikoff CP, Kinsey JA, Braun EL, Zelter A, Schulte U, Kothe GO, Jedd G, Mewes W, Staben C, Marcotte E, Greenberg D, Roy A, Foley K, Naylor J, Stange-Thomann N, Barrett R, Gnerre S, Kamal M, Kamvysselis M, Mauceli E, Bielke C, Rudd S, Frishman D, Krystofova S, Rasmussen C, Metzenberg RL, Perkins DD, Kroken S, Cogoni C, Macino G, Catcheside D, Li W, Pratt RJ, Osmani SA, DeSouza CPC, Glass L, Orbach MJ, Berglund JA, Voelker R, Yarden O, Plamann M, Seiler S, Dunlap J, Radford A, Aramayo R, Natvig DO, Alex LA, Mannhaupt G, Ebbole DJ, Freitag M, Paulsen I, Sachs MS, Lander ES, Nusbaum C, Birren B (2003). The genome sequence of the filamentous fungus *Neurospora crassa*. Nature.

[25] Nowrousian M, Stajich J, Chu M, Engh I, Espagne E, Halliday K, Kamerwerd J, Kempken F, Knab B, Kuo HC, Osiewacz HD, Pöggeler S, Read N, Seiler S, Smith K, Zickler D, Kück U, Freitag M (2010). De novo Assembly of a 40 Mb Eukaryotic Genome from Short Sequence Reads: *Sordaria macrospora*, a Model Organism for Fungal Morphogenesis. PloS Genetics.

[26] Esser K (1982). Cryptogams-Cyanaobacteria, Fungi, Algae and Lichens.

[27] Straus DC (2009). Molds, mycotoxins, and sick building syndrome. Toxicol. Ind. Health.

[28] Froehner SC, Eriksson K-E (1974). Purification and properties of *Neurospora crassa laccase*. J. Bacteriol.

[29] Minuth W, Esser K, Klischies M (1978). The phenoloxidases of the ascomycete *Podospora anserina*. Eur. J. Biochem.

[30] Fernández-Larrea J, Stahl U (1996). Isolation and characterization of a laccase gene from *Podospora anserina*. Mol. Gen. Genet.

[31] Germann UA, Müller G, Hunziker PE, Lerch K (1988). Characterization of two allelic forms of *Neurospora crassa* laccase. Amino- and carboxyl-terminal processing of a precursor. J. Biol. Chem.

[32] Altschul SF, Madden TL, Schaffer AA, Zhang J, Zhang Z, Miller W, Lipman DJ (1997). Gapped BLAST and PSI-BLAST: a new generation of protein database search programs. Nucleic Acids Res.

[33] Thompson JD, Gibson TJ, Higgins DG (2002). Multiple sequence alignment using ClustalW and ClustalX. Curr. Protoc. Bioinformatics.

[34] Page RD (1996). TreeView: an application to display phylogenetic trees on personal computers. Comput. Appl. Biosci.

[35] Germann UA, Lerch K (1986). Isolation and partial nucleotide sequence of the laccase gene from *Neurospora crassa*: amino acid sequence homology of the protein to human ceruloplasmin. Proc. Natl. Acad. Sci. USA.

[36] Giardina P, Faraco V, Pezzella C, Piscitelli A, Vanhulle S, Sannia G (2010). Laccases: a never-ending story. Cell Mol. Life Sci.

[37] Emanuelsson O, Brunak S, von Heijne G, Nielsen H (2007). Locating proteins in the cell using TargetP, SignalP and related tools. Nat. Protoc.

[38] Tusnády GE, Simon I (1998). Principles governing amino acid composition of integral membrane proteins: application to topology prediction. J. Mol. Biol.

[39] Tusnády GE, Simon I (2001). The HMMTOP transmembrane topology prediction server. Bioinformatics.

[40] Kumar SVS, Phale PS, Durani S, Wangikar PP (2003). Combined sequence and structure analysis of the fungal laccase family. Biotechnol. Bioeng.

[41] Hoegger P, Navarro-González M, Kilaru S, Hoffmann M, Westbrook E, Kües U (2004). The laccase gene family in *Coprinopsis cinerea (Coprinus cinereus)*. Curr. Genet.

[42] Velázquez-Cedeño M, Farnet A, Billette C, Mata G, Savoie J-M (2007). Interspecific interactions with *Trichoderma longibrachiatum* induce *Pleurotus ostreatus* defence reactions based on the production of laccase isozymes. Biotechnol. Lett.

[43] Askwith C, Eide D, Van Ho A, Bernard PS, Li L, Davis-Kaplan S, Sipe DM, Kaplan J (1994). The FET3 gene of *S. cerevisiae* encodes a multicopper oxidase required for ferrous iron uptake. Cell.

[44] Stearman R, Yuan DS, Yamaguchi-Iwai Y, Klausner RD, Dancis A (1996). A permease-oxidase complex involved in high-affinity iron uptake in yeast. Science.

[45] Messerschmidt A, Huber R (1990). The blue oxidases, ascorbate oxidase, laccase and ceruloplasmin Modelling and structural relationships. Eur. J. Biochem.

[46] Pignocchi C, Foyer CH (2003). Apoplastic ascorbate metabolism and its role in the regulation of cell signalling. Curr. Opin. Plant Biol.

[47] Koikeda S, Ando K, Kaji H, Inoue T, Murao S, Takeuchi K, Samejima T (1993). Molecular cloning of the gene for bilirubin oxidase from *Myrothecium verrucaria* and its expression in yeast. J. Biol. Chem.

[48] Mizutani K, Toyoda M, Sagara K, Takahashi N, Sato A, Kamitaka Y, Tsujimura S, Nakanishim Y, Sugiura T, Yamaguchi S, Kano K, Mikami B (2010). X-ray analysis of bilirubin oxidase from *Myrothecium verrucaria* at 2.3 A resolution using a twinned crystal. Acta Crystallogr. Sect. F. Struct. Biol. Cryst. Commun.

[49] Tanaka N, Murao S (1982). Purification and some properties of bilirubin *Myrothecium verrucaria* MT-1. Agri. Biol. Chem.

[50] Pointelli E, Santa-Maria MA, Caretta G (1981). Coprophilous fungi of the horse. Mycopathologia.

[51] Nowrousian M, Würtz C, Pöggeler S, Kück U (2004). Comparative sequence analysis of *Sordaria macrospora* and *Neurospora crassa* as a means to improve genome annotation. Fungal Genet. Biol.

[52] Engh I, Nowrousian M, Kück U (2010). *Sordaria macrospora*, a model organism to study fungal cellular development. Eur. J. Cell Biol.

[53] Pöggeler S (1999). Phylogenetic relationships between mating-type sequences from homothallic and heterothallic ascomycetes. Curr. Genet.

[54] Kempken F (1995). Horizontal transfer of a mitochondrial plasmid. Mol. Gen. Genet.

