# Supplementary material for: Evolution of Multicopper Oxidase Genes in Coprophilous and Non-Coprophilous Members of the Order Sordariales
Source: Curr Genomics. 2011 Apr;12(2):95–103. doi: 10.2174/138920211795564368 (PMC3129052; doi:10.2174/138920211795564368)
Supplement: Supplementary file 1 [file CG-12-95_SD1.pdf]

SUPPLEMENTARY MATERIAL

```
                      *        20         *        40         *        60         *        80         *
SMAC_03279 : GARAWIRVYN-DMSDL--NLTMHWHGLTQRMAPFADGTPQASQWPIPP-GHFFDYEVATETGDAGTYFY-HSHV----AMQATT-CSGPLVVDDC : 85
Pa_4_3640| : GTSSWIRVYN-DMPDQ--NLTMHWHGLTQRMAPFADGTPLASQWPIPP-GHFFDYEIATNNDDAGTYFY-HSHV----DIQAIS-CTGPLIVDDC : 85
CHGG08781. : GARTWIRVYN-DIPDQ--NLTMHWHGLSQRFAPFADGTLGASQWPIPP-GHFFDYEIATELEDAGSYFY-HSHV----GLQALS-CTGPLIVEDC : 85
SMAC_07233 : GDRLVVNLLN-SLGDE--DTSLHFHGLFMNGSTHMDGASMVSQCPIPR-GASFTYDFTID--QPGTYWY-HSHT----QGQYPDGFRGPFIIHDP : 84
NCU03498.4 : GDRLIVNLHN-SLGDE--DTSLHFHGLFMNGSNHMDGPVMVTQCPIPP-GASFTYNFTVD--QPGTYWY-HSHN----KGQYPDGFRGPLVIHDP : 84
CHGG08215. : GDRLVVNVFN-SLGNQ--STSLHFHGLFMSGATHMDGPVSVTQCPIVP-GGRFTYNFTVD--QPGTYWY-HSHI----HGQYPDGLRGPLIVHDP : 84
Pa_6_4220| : GDRVIVHLLN-LLETE--PTSLHFHGLYLRNETHMDGPVHVSQCTIPP-GSSFTYNFTVN--EPGIYWY-HSHV----HGQYPDGLRGPLIIHDP : 84
SMAC_02760 : GDTLIVNLHN-GLGNQ--TTGLHWHGINQLQTPEMDGPSGVVQCPTPP-GSTVQYKFLLD--EPGTFWY-HSHE----KGQYPDGLRGPLIVQDP : 84
NCU04593.4 : GDTLIVNLHN-GLGNQ--TTGIHWHGINQLQTPEMDGPSGVVQCPTPP-GSSVQYKFVLD--EPGTFWY-HSHE----KGQYPDGLRGPLIVQDP : 84
Pa_2_530|c : GDTVIINFTN-NLGNQ--TSGLHFHGINQVQTPEMDGPSGVTQCPVPP-GSTLQYKFVVD--VGGTFWY-HSHN----MGQYPDGLRGVFLVHDP : 84
CHG00543.1 : GDTVVVNLNN-KLDNQ--TTGLHFHGMNQVQTSDMDGPSGVTQCPIPP-NSSVKYQFTAD--APGTYWYDHSHN----MGQYPDGLRGSLIVQDP : 85
SMAC_01222 : GDKIVVNVFN-NISSPEEGTSIHWHGFLHQDTPWDDGAPGVSQCPIAP-GKSYTYEFKAS--LYGTSWY-HAHY----SAQYAGGIVGPIVVHGP : 86
NCU00526.4 : GDMIVVNVFN-NISSPEEGTAMHWHGFLQDDTPWDDGAPGVSQCPIVP-GKSYTYEFNAS--LYGTSWY-HAHY----SAQYAGGIVGPIVVHGP : 86
CHGG06172. : GDTIIVNVHN-NITGPEDGTAIHWHGFLQHETPWEDGAPGISQCPIPP-RKSYSYEFIAS--LYGTSWY-HAHY----SAQFAGGILGPIVVHGP : 86
Pa_1_15470 : GDMIVVNVRN-NITNPEDGTAIHWHGFLQTETPWEDGAPGISQCPIPP-GTSYRYEFLAS--LYGSTWY-HAHY----SAQYAGGIVGPMVIHGP : 86
SMAC_09572 : GDWIEVTLVN-KIKDPGEGAAIHWHGIRQVGTPWMDGVPSTTQCPIPP-GHRFTYRFRAD--EYGSSFW-HSHV----GSQYSAGAFGAIVVHGP : 86
SMAC_03641 : GDSLVIHVTN-NLKYN--GTAIHWHGLRQSGSNIYDGVPGITQCAIAI-GQSMTYRFKVT--QYGSSWY-HSHF----SLQYANGVFGGIVFNGP : 84
NCU02201.4 : GDSLIIHVTN-NLKSN--GTGIHWHGLRQSGSNIYDGVPGITQCAIAI-GQSMTYRFKVS--QYGSTWY-HSHF----SLQYANGVFGGMIFNGP : 84
CHGG11082. : GDEVIIHVTN-NMASN--GTSIHWHGLRMLNNALNDGVPGVTQCGLAP-GETMTYRFRVT--QYGSTWY-HSHF----SLQYAEGLFGGMILRGP : 84
Pa_5_9860| : GDDLVIHVTN-NMLEN--GTAIHWHGLHMRDNSLNDGVPGVTQCGIRP-GDTQTYRFKVT--QYGSTWY-HSHF----SLQYAEGLFGGMIFNGP : 84
SMAC_09228 : GDTVIVHVTN-SLDDE--GTVIHWHGLRQYYSNDQDGV-ALTQCPIAR-GSTWTYNFTML--QYGTTWY-HSHY----SVQYADGVLGPLVIHGP : 83
NCU09023.4 : GDTVIVHVTN-NLDDE--GSVIHWHGLRQFYNNDQDGV-ALTQCPIAR-GSTWTYNFTML--QYGTTWY-HSHY----ILQYADGVLGPVVVHGP : 83
CHGG_10025 : GDELVIHVTNNDHFGN--GTTVHWHGMRQLNTNQMDGVNGITQCPIAP-GDTFTYRFRAT--QYGHSWY-HSHY----SMQYADGVVGPLVIHGP : 85
Pa_5_4140| : GDELVITVKN-SLPNM--GTQIHWHGIRQLFTNDMDGV-AVTQCPIAR-GHTFQYKFRVL--QYGSTWY-HSHY----SLQYSDGLCGPLVIHGP : 83
Pa_7_3560| : GDKIVVHVRN-KYRDN--GTTIHWHGLRQLYSNDMDGVNGVTQCPIAY-GEEYTYEFRAR--QYGHTWY-HSHY----SLQYPDGVAGPLIIHGP : 84
SMAC_06098 : GDYLEITVIN-RLKSN--GTSIHWHGMHQRNTNIHDGVNGVTECPIPPNGGSKVYRFRAT--QYGTSWY-HSHF----SAQYGNGIVGSIVINGP : 85
NCU04528.4 : GDYIEITVIN-KLKSN--GTSIHWHGMHQRNSNIQDGVNGVTECPIPPRGGSKVYRWRAT--QYGTSWY-HSHF----SAQYGNGIVGPIVINGP : 85
CHGG02290. : GDTIQVTVIN-NLDTN--GTSIHWHGMHQKDTNLHDGANGVTECPIPP-GGRRVYRFKAQ--QYGTSWY-HSHF----SAQYGNGVVGTIQINGP : 84
Pa_5_1200| : GDWIQVNVIN-NLRTN--GTSIHWHGLHQKGTNMHDGANGVTECPIPPKGGSRIYRFRAQ--QYGTSWY-HSHF----SAQYGNGVVGTIVVNGP : 85
NCU05113.4 : GDRIEVTVTN-KLKTN--GTSMHWHGFHQLNSNNQDGVAGVTECLIVP-GASKVYSFLAT--QHGTSWY-HSHI----SSQYANGIVGPIQINGP : 84
Pa_5_4660| : GDRINVTVVN-NLVSN--GTSIHFHGIHQKNTNNNDGVSGVTECPIAP-GRSKTYSFIAT--QYGTAWY-HSHF----SAQYGNGVLGAIRINGP : 84
SMAC_08663 : GDRLQITVIN-NMRSN--GTSIHYHGLRMLNANTQDGVNGVTECPIPP-GSRKTYTFLAN--QYGTAWY-HSHF----PLQYGNGILGPIVIKGP : 84
NCU09279.4 : GDRLQITVIN-NMRSN--GTSIHYHGLRMLNANTQDGVNGVTECPIPP-GSRKTYTFLAN--QYGTAWY-HSHF----PLQYGNGVLGPIVIKGP : 84
SMAC_03318 : GDWINVTVIN-KLPAN--GTSIHWHGIRMLNNNINDGVNGITECPIPP-NGTKSYVFLAQ--QYGTTWY-HSHH----SSQYGNGVFGPLVINGP : 84
NCU05604.4 : GDYVNVTIIN-KLLYN--GTSIHWHGLQMEDNNINDGVGGVTECPIPP-NATKSYLFLAE--QYGTTWY-HSHY----SAQYGNGVLGAIVFNGP : 84
CHGG035521 : GDYIVVNVYN-DMQDN--GTSIHWHGIRQFGESNQDGANGVTECPIPP-GHMKTYDFHVT--QYGTSWY-HSHY----SNQYGNGVVGSLVVRGP : 84
Pa_7_4200| : GDYIIVNVYN-DMQDN--GTSIHWHGIRQYGESNQDGANGVTECPIPP-GSMKTYDFHVT--QYGTSWY-HSHY----SNQYGNGVVGPLIVRGP : 84
SMAC_09326 : GDYVQVNVYN-DLQHN--GTSIHWHGIRQFGESNQDGANGVTECPIPP-GSMKSYTFHVT--QYGTSWY-HSHY----SNQYGNGVVGALIVKGP : 84
SMAC_08961 : GDDVEVKVCN-HLPDN--GTNIHWHGIRQFYTNYADGATAQTECPIAP-GDCHTYKWKAT--QHGSSWY-HSHY----SIQYSEGLLGPIIIHGP : 84
SMAC_09721 : GDDVEVKVCN-HLPDN--GTSIHWHGIRQFYTNYADGATSQTECPIAP-GDCHTYKWKAT--QHGSSWY-HSHY----SIQYSEGLLGPIIIHGP : 84
Pa_6_10630 : GDSVQVEVCN-NLSQN--GTSIHFHGVRHLNTNHADGAVSQTECPIAP-GDCHTYRFKAT--QHGSSWY-HSHY----SLQYGDGLLGAIVIHGP : 84
SMAC_03042 : GDWLEVTVKN-NLRWN--GTSVHWHGFRQLESFHMDGVNGITECPIAP-GDTFTYRFRAM--QYGSAWY-HSHY----SLQYGDGLLGPITIYGP : 84
NCU07920.4 : GDWIEVTVKN-NLRWN--GTSVHWHGFRQFESFHMDGVNGITECPIAP-GDTFTYRFRAM--QYGSAWY-HSHY----SLQYGDGLLGPITIYGP : 84
Pa_6_7880| : GDTIEVTIKN-NLRWN--GTSVHWHGIRQWETFQFDGVNGITECPIAP-GETFTYRFRAL--QYGSAWY-HSHY----SLQYGDGLLGPITIYGP : 84
Pa_6_2550| : GDIVRVKVNN-LIWDE--STTIHWHGIHQRNTTWMDGVAGISQCAIPP-GKSFTYEFEIID-QRGTFWY-HAHS----KVQYTDGLYGPIVVHDP : 85
SMAC_07604 : GTESVVRFVN-NGRLA---NSVHLHGSFSRAP-----FDGWAEDITPP-GWYKDYYYPNSQ-AARTLWY-HDHAIDHTAENAYYGQAGAYILHDP : 83
NCU05042.4 : GTETVVRFVN-NARLA---NSVHLHGSYSRAP-----FDGWAEDITPP-GWYKDYYYPNSQ-SGRTLWY-HDHAIDHTAENAYYGQAGAYILHDP : 83
Pa_6_11170 : GHEAVVRFVN-NASIE---NSVHLHGSYSRAP-----WDGWAEDVTMP-GEFKDYYYPNQQ-AARFLWY-HDHAFMHTAENAYFGQAGAYIIHDP : 83
Pa_5_1710| : GTESVVRFVN-NAHAN---SSIHLHGSYSRAP-----FDGWAEDTTAP-GEYKDYYFPNRQ-SGRMMWY-HDHA---TAENAYMGQAGVYLVCDP : 80
             Gd   6   N           6H HG         dg     2c   p g    Y         g  55 HsH       q   g  G      p
```

Fig. (S1). Clustal X Alignment of MCOs from Neurospora crassa, Sordaria macrospora, Chaetomium globosum and Podospora anserina. Alignment includes amino acid region of laccase L1 and L2 signature sequences.
